# Proximal connection anomalies of the coronary arteries to the aorta: retrospective experience in a Moroccan university hospital and literature review

**DOI:** 10.11604/pamj.2024.48.139.42943

**Published:** 2024-07-29

**Authors:** Nouhaila Lahmouch, Abdelmajid Bouzerda, Ilyasse Asfalou, Zouhair Lakhal, Aatif Benyass

**Affiliations:** 1 Department of Cardiology, Mohammed V Military Hospital, Faculty of Medicine and Pharmacy of Rabat, Mohammed V University, Rabat, Morocco,; 2 Department of Cardiology, Avicenne Military Hospital, Faculty of Medicine and Pharmacy, Cadi Ayad University, Marrakech, Morroco

**Keywords:** Connection anomalies, coronary arteries, computed tomography coronary angiography, myocardial ischemia, sudden death

## Abstract

Proximal connection anomalies of the coronary arteries to the aorta represent a rare pathology, with an angiographic prevalence close to 1%. The prognosis of this condition is contingent upon its anatomical form. Some instances are linked to sudden deaths, while others may be associated with myocardial ischemia. Utilizing computed tomography (CT) coronary angiography as the optimal imaging tool, one can identify the origin and course of the ectopic artery. Current guidelines suggest surgical correction as the primary intervention for symptomatic abnormalities when a risky form is identified. To enhance the management of these anomalies, the establishment of comprehensive multicenter observational registers is imperative. Our study, a retrospective analysis spanning two years, focuses on 10 cases of proximal connection anomalies of coronary arteries to the aorta diagnosed in the Cardiology department of the Mohammed V Military Hospital of Rabat. By presenting this series and conducting a literature review, we elucidate the anatomical, epidemiological, physiopathological, clinical, angiographic, and CT angiography features of these anomalies, along with insights into therapeutic management.

## Introduction

Congenital coronary anomalies (CCAs), also known as abnormal coronary connections (ACC), involve the anomalous connection of a matured coronary artery to the aorta. The preference for the term “connection” arises from its reflection of the developmental nature of coronary arteries, indicating their attachment to the aorta rather than originating directly from it. These anomalies are infrequent, affecting approximately 0.1 to 0.3% of the general population [1]. Diagnosis is often fortuitous, but certain anatomical variations can manifest with ischemia symptoms. Failure to recognize this anomaly may lead to severe consequences, including 15 to 20% of sudden deaths in young individuals during intense physical exertion [2].

Our study, a retrospective analysis spanning two years, focuses on 10 cases of proximal connection anomalies of coronary arteries to the aorta diagnosed in the Cardiology department of the Mohammed V Military Hospital of Rabat. This study provides insights into the demographic and clinical characteristics of patients with proximal connection anomalies of coronary arteries to the aorta. The study underscores the need for individualized therapeutic management and multidisciplinary collaboration in treating patients with abnormal coronary connections.

## Methods

We conducted a retrospective case series analysis to examine 10 cases involving proximal connection anomalies of the coronary arteries to the aorta. These cases were identified by the Cardiology Department of the Mohammed V Military Hospital in Rabat over two years. Patients included in this study were diagnosed with proximal connection anomalies of coronary arteries to the aorta. They were selected based on standardized diagnostic criteria, and cases with incomplete medical records or missing diagnostic imaging were excluded from the analysis. Data for this study were gathered from patient medical records, diagnostic reports, angiographic images, and CT angiography scans. We examined various aspects, including demographic information such as age and sex, clinical features, and detailed findings from angiographic and CT imaging related to coronary anomalies. The sample size of 10 cases was determined by the number of eligible patients available during the study period. Due to the small sample size and the retrospective nature of the study, we used descriptive statistics to summarize the data, focusing on presenting the demographic and clinical characteristics as well as documenting the specific details of each coronary anomaly. No inferential statistical analysis was performed. It is important to note that biases in case series studies can be challenging to completely avoid due to the retrospective design and limited sample size.

## Results

**Demographics:** the patients' ages ranged from 50 to 68 years, with a median age of 58.4 years, and a predominant 80% male involvement. Cardiovascular risk factors were heavily skewed toward active smoking, which was exclusively observed in males, constituting 70% of cases, followed by hypertension at 60%.

**Clinical presentation:** the predominant symptom among our patients was chest pain, observed in 90% of cases. This pain was further classified as infarct pain in 40% of cases, effort angina in 40%, and rest angina in 10%. In the remaining 10%, the chief complaint was exertional dyspnea, which correlated with tight mitral stenosis.

**Discovery of anomalies:** the abnormal coronary connections (AAC) in our patients were incidental findings during coronary angiography prompted by various clinical presentations. Among these presentations, there were four acute coronary syndromes: two cases of ST-segment elevation myocardial infarction (STEMI) and two cases of non-ST-segment elevation myocardial infarction (NSTEMI). Additionally, two patients presented with effort/rest angina. The other cases involved uncomplicated post-myocardial infarction, ischemic heart disease in the dilated stage, dilated non-ischemic cardiomyopathy, and mitral stenosis requiring surgical intervention. In 60% of our patients, atherosclerotic coronary involvement was detected, thus revealing the proximal connection anomaly of the coronary artery.

**Imaging and identification of coronary anomalies:** all patients underwent trans-thoracic echocardiography, but none of the examinations raised suspicions of coronary connection anomalies. The diagnosis was ultimately established through coronary angiography for all patients. Complementary cardiac computed tomography angiography (CCTA) was performed to better understand the origin and pathway of the anomaly.

**Figure 1:**
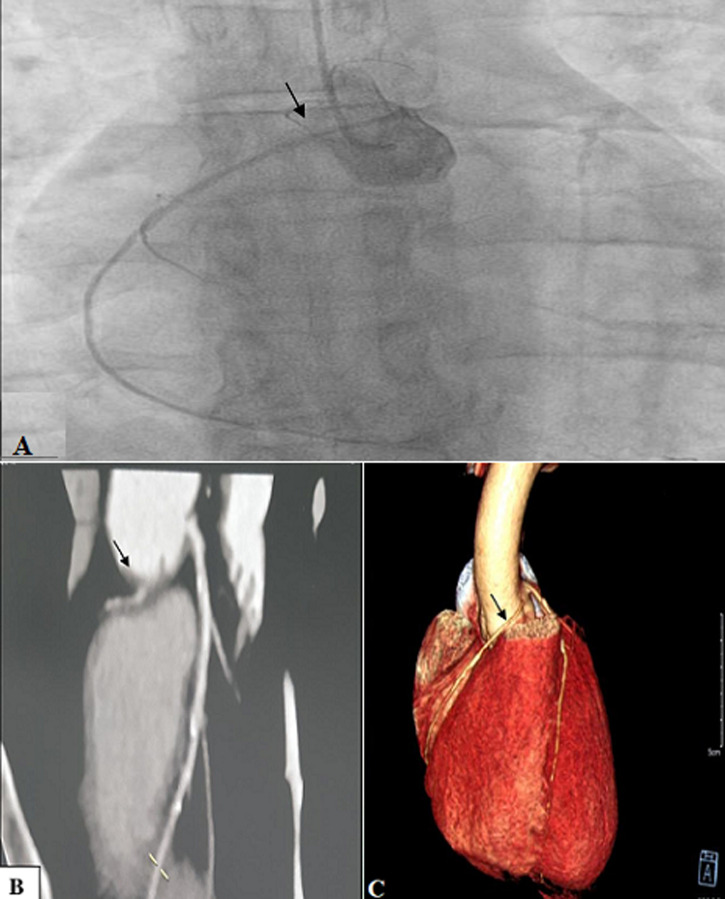
patient #2; (A) angiographic image depicting an ectopic connection of the right coronary artery to the left sinus (arrow); (B, C) CT images showing an unusual connexion of the right coronary artery 7.3mm in front of the birth of the common trunk (arrow) with a proximal inter aorto-pulmonary path

**Figure 2:**
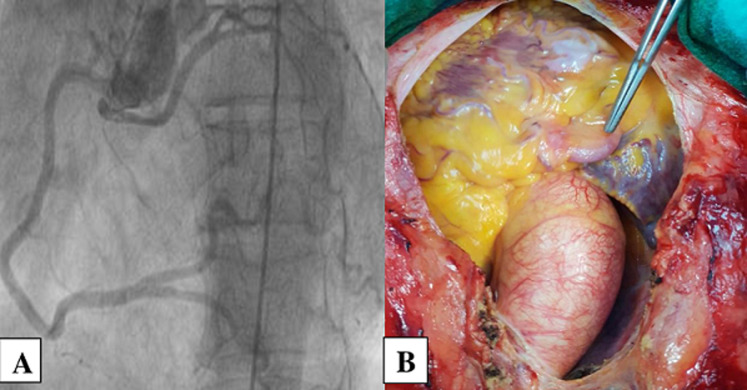
patient #7; A) angiographic image; B) a perioperative view of an ectopic origin of the left coronary artery from the antero-right sinus

**Types of coronary anomalies:** the right coronary artery was the most common site of anomaly, occurring in 60% of cases. The circumflex artery and the left coronary artery each accounted for 20% (2 cases). The right coronary artery connected to the left main coronary artery in 2 cases, the antero-left sinus in 3 cases (Figure 1A, B patient #2), and the junction of the left and non-coronary sinus in 1 case. Other anomalies involved the circumflex artery connected to the right sinus (1 case) (Figure 2, patient #7) and the left coronary artery connected to the right sinus (3 cases).

**Figure 3:**
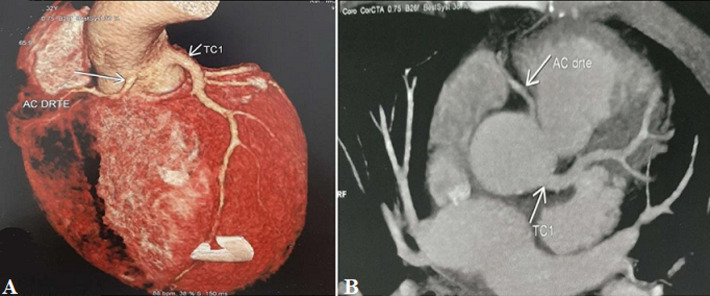
A, B); patient #6 CT coronary images showing the connexion of the right coronary artery to the anteroleft sinus almost 20mm medial of the birth of the left coronary artery with an initial retro-aortic course (A, B)

**Figure 4:**
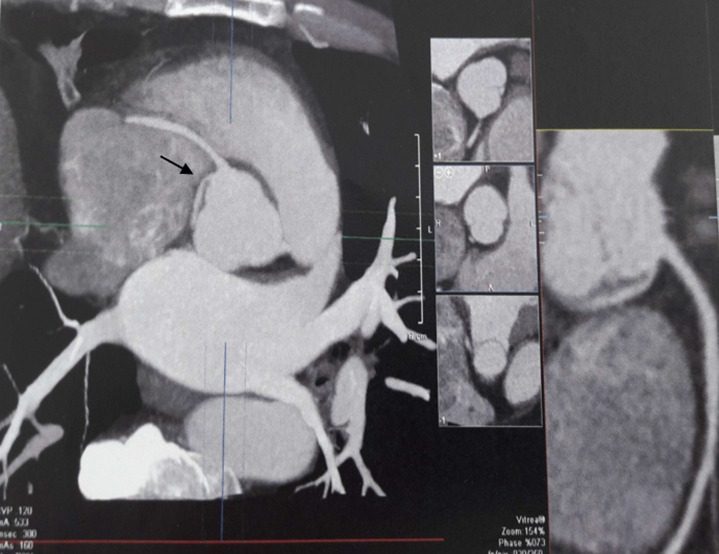
patient #8 CT coronary angiography revealing the connexion of the circumflex artery to the right sinus with a proximal path between the aorta and the right atrium (arrow) without an intramural segment

**Pathways of the anomalies:** regarding the type of coronary anomaly, connections to the contralateral sinus were the most frequent, accounting for 80% of anomalies (8 cases), while connections to the contralateral artery represented 20% (2 cases). The proximal pathway of the anomaly was studied in 50% of cases using CCTA, revealing an inter-aorto-pulmonary pathway for the right coronary artery (Figure 1B) in 30% of cases (3 cases) and a retroaortic pathway in 10% of cases (1 case) (Figure 3, patient #6). The circumflex artery exhibited a retro-aortic pathway in one case (Figure 4, patient #8).

**Figure 5:**
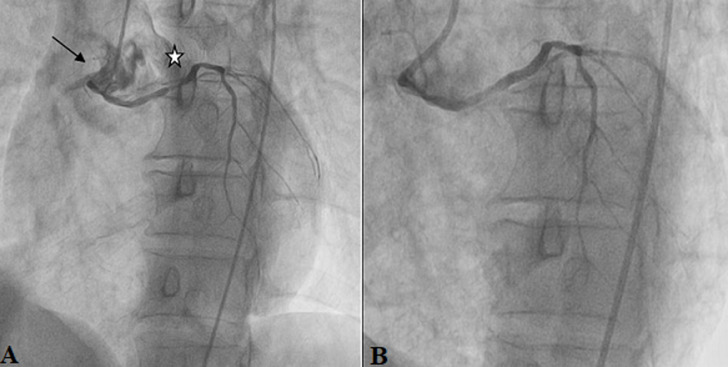
A, B); patient #4 angiographic images of an ectopic connection of the left coronary artery with the antero-right sinus (black arrow) presenting significant stenosis of 70-90% (star) treated by angioplasty with placement of a drug eluted stent

**Treatment and outcomes:** conventional medical treatment was prescribed for all patients based on their clinical presentation. One patient with effort/rest angina and a normal coronary angiography, except for a circumflex artery connected to the antero-right sinus, received beta-blocker treatment. Primary angioplasty with drug-eluting stent placement was performed in 75% of ischemic cases (Figure 5, patient #4). One patient underwent mitral valve replacement with a mechanical prosthesis, and two patients with triple-vessel coronary lesions, one of whom had a right coronary artery with an inter-aorta-pulmonary pathway, underwent triple coronary artery bypass surgery. Post-operative care for the operated patients was uneventful, with no complications during hospitalization, and they recovered well. None of the patients required readmission after discharge. All results are summarized in Table 1.

**Table 1:** summary of clinical cases

Patient	Age, sexe	Cardiovascular Risk Factors	Clinical presentation	Coronary angiography+/- coronary CT scann	Treatment
1	**50, Female**	- Diabetes - Hypertension	**Inferior ST-elevation myocardial infarction STEMI**	- Right coronary artery connected to the left main artery - Significant lesion in the mid-circumflex artery	Primary angioplasty of the mid-circumflex artery with drug eluted stent
2	**55, Male**	- Smoking - Hypertension	**Dilated cardiomyopathy LVEF=35%**	- Right coronary artery connected to the left main artery - Non significant lesion in the mid-LAD	Medical treatment
3	**65, Male**	- Smoking	**Non-ST-elevation myocardial infarction NSTEMI**	- Right coronary artery connected to the sino-tubular junction of the left sinus with a 20 mm inter-aortopulmonary course - Significant stenosis of the proximal LAD	Primary angioplasty of the proximal LAD with a Drug eluted stent
4	**59, Male**	- Diabetes - Hypertension - Dyslipidemia - Smoking	**Effort angina**	- Left main coronary artery network connected to the right sinus - Significant stenosis of the mid-LAD - Significant stenosis in the right coronary artery	Aorto-coronary bypass
5	**58, Male**	- Smoking - Dyslipidemia	**Non-ST-elevation myocardial infarction NSTEMI**	- Right coronary artery connected to the antero-left sinus -Sub-occlusive stenosis of the marginal artery	- Primary angioplasty of the proximal marginal artery with drug eluted stent
6	**52, Male**	- Diabetes - Obesity	**Dilated cardiomyopathy LVEF=25%**	- Right coronary artery connected to the antero-left sinus - Normal coronary angiography	Medical treatment
7	**55, Female**	-Hypertension	**Surgical repair of severe mitral stenosis**	- Left main coronary artery connected to the right sinus	Surgical mitral valve replacement
8	**63, Male**	-Hypertension - Smoking	**Effort and rest angina**	- Normal coronary angiography - Circumflex artery connected to the antero-right sinus with retro-aortic pathway	Medical treatment
9	**59, Male**	- Smoking - Obesity	**Antero-septal ST-elevation myocardial infarction STEMI**	- Significant stenosis of the mid-circumflex artery connected to the right coronary artery -Occlusion of the proximal LAD	- Primary angioplasty of the proximal LAD and the circumflex with drug eluted stent
10	**68, Male**	-Smoking - Hypertension	**Inferior post myocardial infarction**	- Right coronary artery connected to the antero-left sinus with severe stenosis - Significant stenosis in mid and distal LAD - Significant stenosis of the first marginal artery	Triple aorto-coronary bypass

## Discussion

Proximal connection anomalies of the coronary arteries are relatively rare, with an angiographic prevalence ranging from 0.6% to 3% in various studies [3-6]. Although these anomalies predominantly involve the left coronary artery trunk, they have been reported across the entire coronary network, exhibiting a computed tomography angiography (CCTA) prevalence of 1.3% in adult populations. The formation of coronary arteries involves a complex process during embryonic development. Sinusoids develop within the embryonic myocardium, while epicardial blood islets coalesce to form a rudimentary plexus. The final coronary network is shaped through a series of developmental stages, with coronary ostia joining the aorta at corresponding Valsalva sinuses [7,8]. In normal hearts, coronary arteries originate from the upper half of the aortic sinuses, typically near the sino-tubular junction [9]. However, the positioning of coronary orifices can vary, leading to proximal connection anomalies where the origin of an artery is in an atypical position relative to other sinuses, coronary arteries, or vessels.

There are two main types of proximal connection anomalies: those with a usual angulation greater than 45 degrees, and those with a very acute angle less than 20 degrees, forming an intramural segment that represents a shared wall between the aorta and coronary artery. Among these anomalies, the most prevalent involves the circumflex artery, with an incidence of 3 cases per 1000, followed by the right coronary artery at 1 case per 1000, and the left coronary artery at 2 cases per 1,000 [10]. The impact of these anomalies can range from benign to potentially life-threatening, depending on the location and the course of the abnormality. To describe these anomalies, we used a simplified five-type classification [11]: i) type 1: abnormal connection to the appropriate sinus; ii) type 2: abnormal connection to the contralateral sinus; iii) type 3: abnormal connection to the contralateral artery; iv) type 4: abnormal connection to the non-coronary sinus; v) type 5: single coronary artery.

The most common anomaly is the connection of a coronary artery to the contralateral sinus, with a proximal segment coursing between the aorta and the pulmonary artery. This course can be within the aortic wall, leading to functional stenosis during exertion due to aortic distension and organic stenosis through intimal reshaping. The interatrial course carries the highest risk, with potential consequences like sudden death due to acute myocardial infarction or arrhythmias caused by coronary artery compression. Other aberrant pathways include retro-aortic, pre-aortic, and pre-pulmonic routes, which generally do not have significant clinical consequences. Diagnostic methods for coronary anomalies focus on detailed imaging. Coronary angiography is the most widely used method for studying coronary artery anatomy and diagnosing anomalies. Complementary techniques like intravascular ultrasound (IVUS) and coronary CT angiography (CCTA) offer additional insights, especially in detecting and visualizing intramural segments or complex pathways. Stress echocardiography and scintigraphy are useful for identifying ischemia caused by coronary anomalies.

Therapeutic approaches for benign anomalies generally involve conservative management, unless associated atheromatous disease requires revascularization. High-risk anomalies often require surgical correction, typically involving unroofing or aortic enlargement with a patch. Risks associated with these surgical approaches include commissural dysfunction leading to aortic leakage, early thrombosis, and pseudoaneurysm formation. Beta-blockers may be used for anti-ischemic purposes, but their efficacy in this context is unclear. In cases of ischemic symptoms, the potential role of coronary angioplasty should be evaluated in adults over 30 years, as stenting is feasible with minimal periprocedural risk [12]. The most critical consideration is the risk of sudden death, which, although low, requires careful evaluation. Studies suggest that most sudden death events occur between the ages of 10 and 30, though they can happen in older individuals with an annual incidence of around 0.3% for left ACC and 0.015% for right ACC [1]. Warning signs, such as chest pain or syncope, might precede the event but are often downplayed, especially among high-performance athletes.

**Figure 6:**
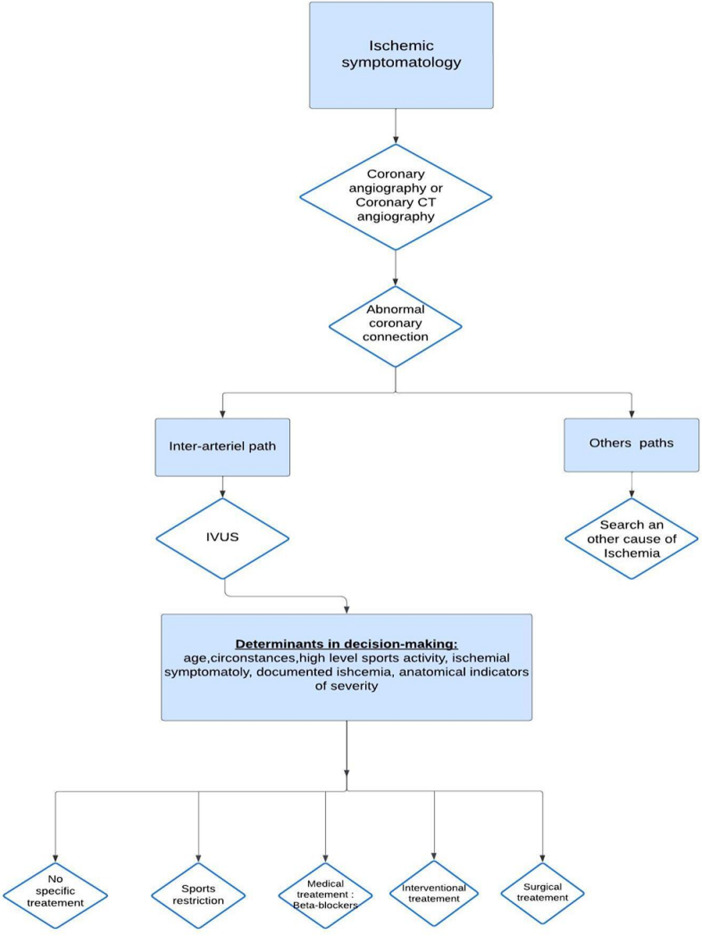
decision tree for aortic abnormal coronary connection (ACC); *IVUS: intravascular ultrasound

The existing recommendations, which are North American and have undergone recent updates [13], suggest that correction is recommended for symptomatic or ischemic coronary anomalies (class I, level of evidence C) and that monitoring is appropriate for asymptomatic cases (class IIa for left ACCs and class IIb for right ACCs, level of evidence C and B respectively). The decision to correct should be made by a specialized multidisciplinary team, with age not being the sole determining factor. Ultimately, successful management of these anomalies relies on accurate diagnosis, careful risk evaluation, and tailored therapeutic strategies. Regular follow-up and ongoing evaluation are critical, especially for patients with high-risk features or prior surgical intervention. The decision algorithm currently employed during ANOCOR group meetings is presented in Figure 6.

## Conclusion

Abnormal coronary connections (ACC) are relatively common, and the prognosis is contingent upon the specific anatomical form. Diagnosis often occurs incidentally, given the substantial number of coronary angiograms, CT scans, and MRIs conducted annually, leading to the discovery of a noteworthy number of these coronary anomalies. When a high-risk form is identified or when symptoms suggestive of ischemia are associated with the anomaly, consideration should be given to correction. The creation of a national registry that consolidates at-risk ACCs, along with their anatomical, clinical, and therapeutic nuances, may prove beneficial in the future by facilitating the delivery of optimal care.

### What is known about this topic

- *Abnormal coronary connections are rare but can lead to significant morbidity and mortality;*
- *Diagnosis often relies on imaging modalities such as coronary angiography and CT angiography;*
- *Therapeutic approaches vary based on the anatomical and clinical characteristics of the anomaly.*

### What this study adds

- *This study provides insights into the demographic and clinical characteristics of patients with proximal connection anomalies of coronary arteries to the aorta;*
- *It highlights the importance of thorough diagnostic evaluation, including coronary angiography and CCTA, in identifying these anomalies;*
- *The study underscores the need for individualized therapeutic management and multidisciplinary collaboration in treating patients with abnormal coronary connections.*
